# Accurate classification of lung nodules on CT images using the TransUnet

**DOI:** 10.3389/fpubh.2022.1060798

**Published:** 2022-12-05

**Authors:** Hongfeng Wang, Hai Zhu, Lihua Ding

**Affiliations:** ^1^School of Network Engineering, Zhoukou Normal University, Zhoukou, China; ^2^College of Public Health, Zhengzhou University, Zhengzhou, China

**Keywords:** lung cancer, computed tomography, lung nodules classification, deep convolutional neural networks, LIDI-IDRI

## Abstract

**Background:**

Computed tomography (CT) is an effective way to scan for lung cancer. The classification of lung nodules in CT screening is completely doctor dependent, which has drawbacks, including difficulty classifying tiny nodules, subjectivity, and high false-positive rates. In recent years, deep convolutional neural networks, a deep learning technology, have been shown to be effective in medical imaging diagnosis. Herein, we propose a deep convolutional neural network technique (TransUnet) to automatically classify lung nodules accurately.

**Methods:**

TransUnet consists of three parts: the transformer, the Unet, and global average pooling (GAP). The transformer encodes discriminative features via global self-attention modeling on CT image patches. The Unet, which collects context by constricting route, enables exact lunge nodule localization. The GAP categorizes CT images, assigning each sample a score. Python was employed to pre-process all CT images in the LIDI-IDRI, and the obtained 8,474 images (3,259 benign and 5,215 lung nodules) were used to evaluate the method's performance.

**Results:**

The accuracies of TransUnet in the training and testing sets were 87.90 and 84.62%. The sensitivity, specificity, and AUC of the proposed TransUnet on the testing dataset were 70.92, 93.17, and 0.862%, respectively (0.844–0.879). We also compared TransUnet to three well-known methods, which outperformed these methods.

**Conclusion:**

The experimental results on LIDI-IDRI demonstrated that the proposed TransUnet has a great performance in classifying lung nodules and has a great potential application in diagnosing lung cancer.

## Introduction

According to the latest statistics, lung cancer remains the leading cause of cancer death worldwide (18.0% of all cancer deaths) (1, 2). Despite the development in diagnosis and treatment, approximately 70% of patients are still diagnosed at the advanced stages, with a 5-year survival rate of only 10 20% (2, 3). Early detection of lung cancer is associated with a better prognosis, increasing the 5-year survival rate to 57% for localized stage disease (4, 5). In recent years, computed tomography (CT) has been proposed for the early detection of lung cancer to improve patient survival and extend life expectancy. It has been shown to reduce mortality by 20-43% and has the advantages of high spatial resolution, cost-effectiveness, and non-invasiveness (6, 7).

Traditionally, the classification of lung nodules as benign or malignant depends entirely on the clinician or radiologist (8). This pattern has some major disadvantages: (1) it is time-consuming and labor-intensive; (2) it requires extensive clinical experience, and even experienced doctors have difficulty in accurately classifying small nodules; and (3) it is subjective and difficult to generalize. As a result, developing a method for the automatic classification of lung nodules is critical. With recent advancements in the field of medicine, the application of artificial intelligence may provide the potential to overcome current obstacles.

Deep learning and machine learning have been able to attain state-of-the-art performance on various tasks in the past 10 years (9, 10), including picture classification, objection detection, and semantic segmentation. In deep learning, deep convolutional neural networks, also known as DCNNs, have demonstrated impressive results in image-processing endeavors (11). Cancer diagnosis using deep learning is also a hot research topic that can assist the clinician in making the right decision (12). For example, Shen et al. proposed a multi-scale convolutional neural network (MCNN) to extract discriminative features of CT images based on stacked layers for lung nodule classification (13). This method is based on deep learning and can automatically learn image features. To improve the performance of deep learning on an imbalanced dataset, Liang et al. proposed a filtering step to remove irrelevant images and reduce the level of imbalance (14). Besides, to improve the detection accuracy of lung nodules, Anirudh et al. developed a 3D CNN for lung nodule detection that can use weakly labeled data to train the network (15). The experimental results were better than the traditional methods.

In contrast to more conventional methods for image classification, such as SVM, logistics, and decision trees, which rely on manually constructed features, the feature extraction process in DCNNs was carried out in a sophisticated manner, owing to the utilization of convolution layers. In addition, upsampling and deconvolution operations were used in most DCNNs to decode the powerful hierarchical feature representation from raw data. Finally, the Softmax layers were used to achieve efficient image classification. Thus, it was crucial to develop a novel DCNNs method to classify the CT image automatically and achieve an intelligent diagnosis of lung cancer.

In this article, we studied the problem of classifying pulmonary nodules in CT images as benign or malignant. Our objective was to develop a method to enhance the precision of intelligent lung cancer diagnosis. To do this, we proposed a new DCNNs method, TransUnet, by exploiting the advantages of deep learning. The TransUnet comprises a transformer that learns abstract features with larger receptive fields for encoding feature representations from input CT images. With the global context modeled in the transformer, a simple decoder called Unet was used to mine the general features of CT images.

Meanwhile, the global average pooling was introduced to make a decision, such as whether the lung nodules are benign or malignant, based on the above feature. Finally, we conducted experiments on a popular, published lung dataset, LIDI-IDRI, to verify the effectiveness of the proposed TransUnet. Furthermore, the findings of the experiments indicate that the suggested TransUnet system can accomplish high-quality categorization of lung nodules with an accuracy of 84.62%, a sensitivity of 93.17%, and a specificity of 70.92%.

## Methods

The LIDC-IDRI (https://wiki.cancerimagingarchive.net/display/public/LIDC-IDRI) dataset from the Lung Image Database Consortium was used to test the proposed technique approach. The complex steps of image feature extraction in a traditional method can be simplified by inputting the original image.

### Data sets

We used the LIDI-IDRI database in this research; it contained 1,018 patients and is a widely web-accessible resource for evaluating lung cancer classification methods (16–18). Multiple clinical thoracic CT scan images and an XML file were included with each case. The size of the images was 512 × 512 pixels. The XML file details the nodule information, including each nodule's location, boundaries, and malignant level. Four experienced medical professionals contributed this information. The information about nodules contained in the XML indicates that the size of nodules ranged anywhere from 3 to 30 mm.

**Figure 1:**
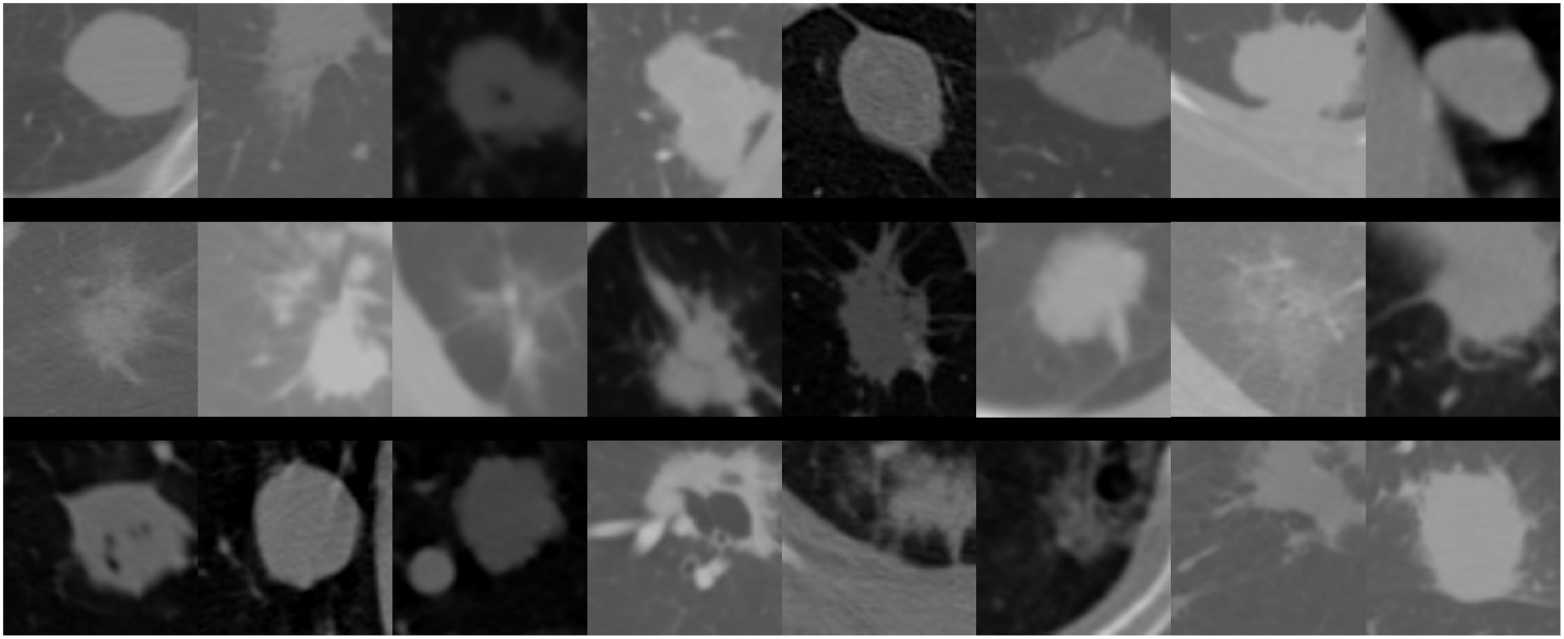
Schematic diagram of lung nodules.

In this study, the location information and the level of lung nodules were obtained using Python. The code is published on https://github.com/mikejhuang/LungNo duleDetectionClassification. In this code, the nodule is classified as benign or malignant according to its level of malignancy. In addition, the images obtained from healthy people are removed from the database. In total, we obtained 8,474 images to evaluate the performance of the proposed method. Some examples of vision CT images are shown in Figure 1.

### TransUnet

**Figure 2:**
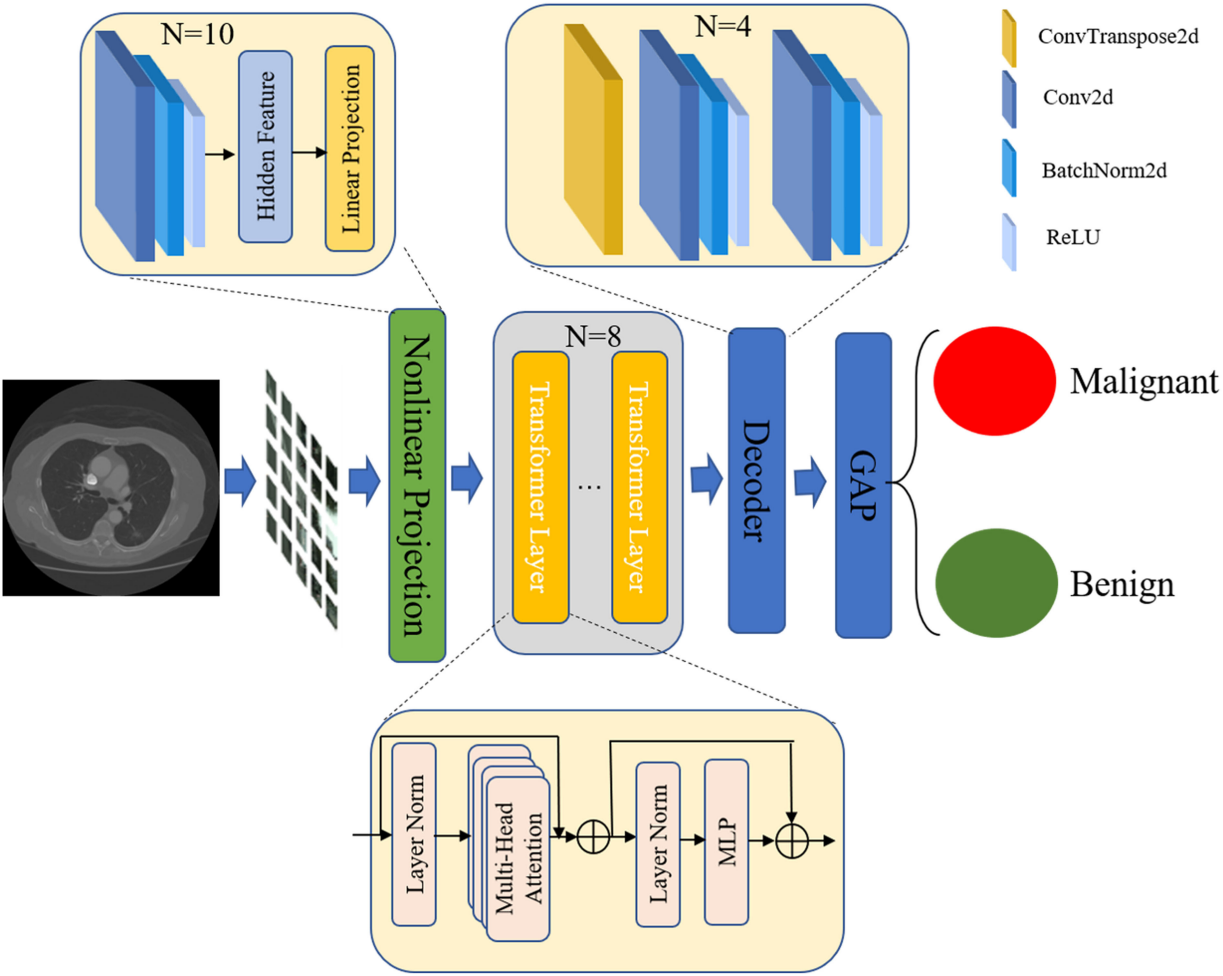
The architecture of TransUnet.

Considering the fact that a nodule is a tiny object in CT images and the background is complex, we proposed a novel TransUnet to identify the nodule level as benign or malignant. TransUnet is based on the transformer and the Unet network. In addition, the structure of TransUnet, as well as the configuration settings, are shown in Figure 2. In particular, the transformer is presented for the purpose of encoding feature representations of input CT scans. Then, the Unet was used to decode the hidden feature for outputting the final classification results.

#### Transformer as encoder

Based on the research by Alexey (19), the first step in the tokenization process involved reshaping the input into a series of flattened 2D patches. Each patch was produced by dividing the CT images used as input into the process. Given a CT image, *x*∈ ℝ^*H*×*W*×*C*^, in which H and W denote the height and width of the image, respectively, and C represents the number of channels. The *x* was divided into small patch *x*_*p*_ that the size is p×p.

Then, the image patch was mapped into embedding space using a trainable nonlinear projection. In this step, we learned about the features of lung nodules by using 10 convolutional layers that have a powerful ability to abstract the general features from CT images. By conducting a number of experiments, we set the convolutional kernel to 2 × 2 and the number of convolutional kernels to 10. In addition, we used the Batchnorm2d and ReLU layers to obtain significant image features. After convolutional layers, a linear projection layer was used to encode the features for generating embedding features. The detailed structure is shown in Table 1.

**Table 1:** Parameter of nonlinear projection.

**Layer**	**Number**	**Output**
Conv2d	10	256×8×8
BatchNorm2d		
ReLU		

Next, we applied a transformer for encoding feature representations from decomposed image patches. The transformer encoder mainly consisted of multi-head self-attention (MSA) (20, 21) and multilayer perceptron (MLP) (22) blocks. As shown in Table 2, the MSA is an extension with multiple independent self-attention operations, and it outputs the lung nodules featured by the concatenated outputs of each self-attention. The MLP block with the residual unit was used to transform the output of MSA. Further, to enhance the learning efficiency, we added two norm layers (23) at the beginning and end of the transformer layer. In this study, considering the computing efficiency, the number of transformer layers was set to 8. After processing multiple transformer layers, we obtained the hidden features of CT images.

**Table 2:** Parameter of the transformer.

**Layer**	**Number**	**Output**
MultiHeadAttention	8	64 × 256
Linear		
PositionalEncoding-42		
PatchEmbedding-43		
LayerNorm-44		
Linear-45		
Dropout-46		
Linear-47		
Dropout-49		
ResidualAdd-50		
LayerNorm-51		
Linear-52		
GELU-53		
Dropout-54		
Linear-55		
MLP-56		
Dropout-57		
ResidualAdd-58		

#### Unet as decoder

In the stage of decoding the feature, as shown in Table 3, we introduced the Unet (24), which comprised numerous upsampling steps to decode the hidden feature and output the final classification results. The Unet network created a path for information propagation between low- and high-level features. The Unet could convert the low-level finer details into high-level semantic features during the training process. In addition, the expansive part could augment the feature by applying up-convolution layers to enlarge the feature maps. The convolution layers were also used in this process to filter the redundant features and obtain the important features for classifying the lung nodules. The up-convolution and convolution operations are utilized alternately, making it possible to create a promising network for semantic segmentation. Considering the advantages of the expansive part of Unet, we used it as our decoder in TransUnet. The output features of the encoder were taken as the input features of a decoder. The decoder consisted of four units for further obtaining general features. In this step, the 2 × 2 up-convolution was used in each unit to halve the number of feature channels and enlarge feature maps. The 3 × 3 convolutions, followed by a ReLU, were used to capture the image feature in enlarged feature maps. Moreover, the BatchNorm was used to normalize the feature distribution for speeding learning. The Relu layer was also applied to restrict the feature and obtain the most highlighted feature. In total, the decoder had 28 layers. In addition, at each upsampling step, the expansive path was used to fuse the multiple-level CT features of the images. The concatenated operation was introduced to fuse the feature map from the contracting path.

**Table 3:** Parameter of the decoder.

**Layer**	**Number**	**Output**
ConvTranspose2d-61	4	2×128×128
Conv2d-62		
BatchNorm2d-63		
ReLU-64		
Conv2d-65		
BatchNorm2d-66		
ReLU-67		

#### Global average pooling for classification

The global average pooling (GAP) (25) in the last network was used to map the general feature to the desired number of classes. The *f*_*k*_(*x, y*) represented the features obtained by unit k in the last convolutional layer at spatial location (x,y). Afterward, the GAP was carried out for unit k by exaggerating the feature at a different position to classify the CT images. The following formula can describe this step:


$$\begin{array}{l} F^{k}=\underset{x,y}{\sum }f_{k}\left(x,y\right), \end{array}$$

Where, the *F*^*k*^ denotes the feature obtained by GAP. The final step is the computation of the category score by the Softmax layer, which is dependent on the findings of the GAP. In particular, the score can be obtained by the following equation:


$$\begin{array}{l} S^{k}=\frac{\exp\left(F^{k}\right)}{\sum_{k}\exp\left(F^{k}\right)}, \end{array}$$

Where, exp denotes the exponential function. In the case of k=0, the *S*^0^ denotes the score of the CT image is considered benign. In the case of k=1, the *S*^1^ denotes the score of the CT image to be malignant.

### Loss function for the deep neural network

We applied cross-entropy (26, 27) to optimize and learn the proposed network parameters. When the network misclassified annotated regions, the cross-entropy tended to give a significant penalty, which guided the network to learn more useful and discriminative patterns. In particular, we trained the proposed TransUnet by attempting to minimize the cross-entropy loss function presented below:


$$\begin{array}{l} L=\underset{k=0,1}{\sum }log\left(S^{k}\right) \end{array}$$

## Results

### Experiment setup

We implemented the proposed method on PyTorch (https://pytorch.org/), a famous open-source platform for deep learning. This study had a total of 8,474 lung CT images of lung nodules acquired from the above LIDI-IDRI. We randomly selected 75% of images (6,355) for training and 25% of images (2,119) for testing. Among the training cases, there were 2,444 benign pulmonary nodules and 3,911 malignant pulmonary nodules. The testing images showed 815 benign pulmonary nodules and 1,304 malignant pulmonary nodules.

We trained our model on a Linux server equipped with Hygon C86 7185 CPUs, 128GB of RAM, and the Sugon DCU. The proposed method was trained until the stable loss was achieved.

### Parameter optimization

The learning rate is one of the important parameters for optimizing deep neural networks. Therefore, we evaluated the performance of the proposed method with different learning rates. The initial learning rate was 0.0001, 0.00001, 0.000001, and was divided by 10 after five epochs decreased using polynomial decay with a power of 0.9. We can see from Table 4 that our proposal achieved the highest accuracy when the learning rate was 0.00001.

**Table 4:** Accuracy and loss value of the proposed method with different optimized parameters.

**Parameter**	**Variation**	**Accuracy**
Learning rate	0.001	0.7220
	0.0001	0.8202
	0.00001	0.7546
Batch size	8	0.7305
	16	0.7546
	32	0.7697
	64	0.7409
Optimizer	Adam	0.7773
	Adadelta	0.7919
	Adagrad	0.8126
	SGD	0.8202

The batch size value was also important to the proposed method's performance. To analyze the effect of batch size, we explored the experiment's various training batch sizes. As shown in Table 4, our method with 16 batch sizes achieved the best performance, which significantly outperformed the other batch size value.

The learning efficiency of the proposed method for great performance also depends on the optimizer. In the case of the same other parameters, the optimizer decides the learning efficiency of the proposed method. As indicated in Table 4, the accuracy of the proposed method was highest when the SGD was used to optimize it.

### Effect evaluation

**Figure 3:**
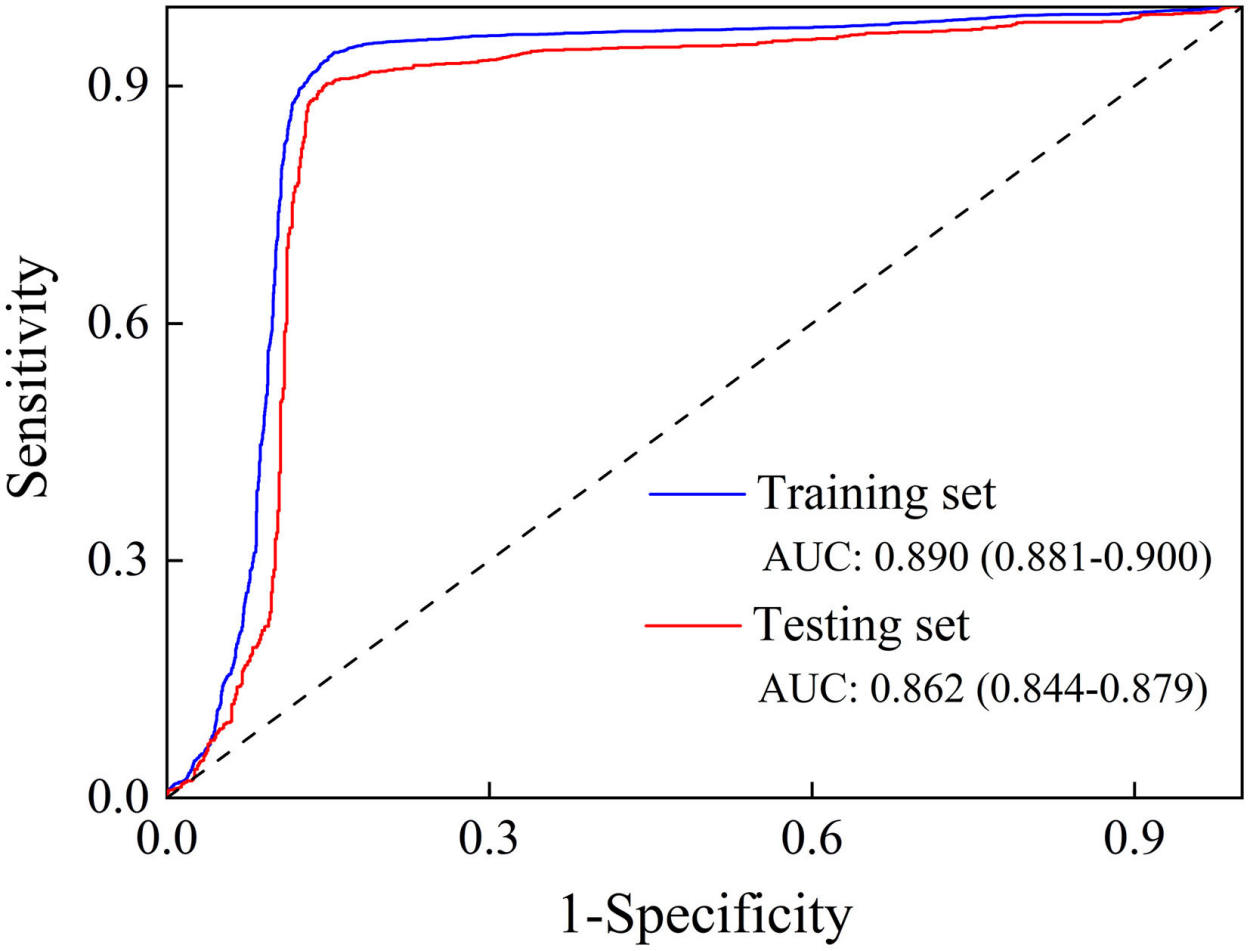
The ROC curve of the training set and the testing set.

We employed sensitivity, specificity, accuracy, and AUC (95% CI) to evaluate the proposed method's performance in classifying pulmonary nodules. As shown in Table 5 and Figure 3, the proposed method achieves 70.92, 93.17, 84.62, and 0.862% (0.844–0.879) in sensitivity, specificity, accuracy, and AUC on the testing set, respectively. In addition, the performance of the proposed method was also evaluated on a training set based on sensitivity, specificity, accuracy, and AUC, and the results obtained were 75.05, 95.93, 87.90, and 0.890% (0.881–0.900), respectively.

**Table 5:** Results of the proposed model to distinguish between benign and malignant lung nodules.

	**Training set**		**Testing set**	
	**Benign lung nodules**	**Malignant lung nodules**	**Benign lung nodules**	**Malignant lung nodules**
Positive	1834	159	578	89
Negative	610	3752	237	1215
Total	2444	3911	815	1304
Sensitivity (%)	75.04		70.92	
Specificity(%)	95.93		93.17	
Accuracy(%)	87.90		84.62	
AUC (95% CI)	0.890 (0.881–0.900)		0.862 (0.844–0.879)	

### Comparison with existing diagnosis methods

We compared the proposed method with the existing advanced methods. The experimental results were reported in the same data set to ensure fairness. The comparison results are shown in Table 6. Song and his colleagues designed a stacked autoencoder (SAE), which is a multilayer sparse autoencoder of a neural network, for the benign and malignant lung nodules (28). Da Silva et al. (29) proposed a network that consists of three convolutional layers and three fully connected layers (29). At the end of each convolutional layer, the ReLu activation was used, and the dropout layer was introduced before the fully connected layer to alleviate the overfitting. Finally, a softmax function was used to classify lung nodules based on the features obtained by the feature extractor. Besides, Kumar et al. (30) proposed a CAD system in which the autoencoder with four layers obtained the feature of images (30). The autoencoder uses a linear or nonlinear transformation to encode the input data into a latent space. Then, the proposed method reconstruct the feature by decoding feature obtained by the decoder.

**Table 6:** Comparison with existing methods.

	**Accuracy (%)**	**Sensitivity (%)**	**Specificity (%)**	**References**
QingZeng Song	82.59	83.96	81.35	(28)
Da Silva	82.3	79.4	83.8	(29)
Kumar	75.01	83.35	N/A	(30)
This work	84.62	70.92	93.17	

The proposed method has achieved the best performance with an accuracy of 84.62%, a sensitivity of 70.92%, and a specificity of 93.17%. The best performance was obtained because the proposed method used the transformer unit to mine potential semantic patterns from multiple image patches. The performance of the proposed method over the other method was significant, with an accuracy and a specificity of at least 2.03% and 9.37%, respectively. For example, compared with the method proposed by Da Silva et al. (29) the proposed method increased by ~2.32 and 9.37% in accuracy and specificity. Good specificity indicates that malignant lung nodules can be diagnosed accurately, which may facilitate the early detection of pulmonary nodules. Finally, based on the comparison results in Table 6, our model was proven to achieve a state-of-the-art level and made some progress in pulmonary nodule diagnosis.

## Discussion

According to the latest GLOBOCAN 2020 statistics, the number of new lung cancer cases worldwide in 2020 was 2,206,771, accounting for 11.4% of all malignant tumor incidences (second only to breast cancer) (2). The number of lung cancer deaths was 1,796,144, accounting for 18.0% of all malignant tumor deaths (the top of all cancers) (2). A new report on the prevalence of malignant tumors in China in 2022 showed that lung cancer ranked first among cancers in terms of both incidence and deaths, with approximately 828,000 and 657,000, respectively (31). Lung cancer is not only a serious threat to the population's health but also an urgent public health problem that increases the burden of the disease (1). Currently, most patients with lung cancer are diagnosed at an advanced stage, and the 5-year survival rate is lower than 20%. Promoting early detection and diagnosis of lung cancer proved to be an effective way to extend the 5-year survival rate and improve the quality of life of patients with lung cancer (7, 32–34).

Nowadays, the “gold standard” for lung cancer diagnosis is a puncture biopsy; however, this is an invasive test and is not widely available in clinical settings (3). Previous studies showed that CT effectively increases lung cancer detection rate and reduces lung cancer mortality (3, 35, 36). For example, in the early twentifirst century, the National Cancer Institute initiated the National Lung Screening Trial, a large randomized controlled study of lung cancer screening that showed that LDCT screening reduced lung cancer mortality by 20.0% (*P* = 0.004) and all-cause mortality by 6.7% (*P* = 0.02) compared with conventional chest x-ray screening (*P* = 0.02) (36–38). However, the classification of lung nodules in CT screening is entirely doctor dependent, which suffers from demerits, including time consumption, difficulty accurately classifying small nodules, being highly subjective, and having high false-positive rates.

To solve these problems, we proposed a novel TransUnet, which combines the transformer and the Unet network to perform the prediction of lung cancer. In a section on parameter optimization, we reported the experimental results with different optimization methods, including learning rate, batch size, and optimizer. The learning rate determines the step for each optimization. When the learning rate is high, the optimization process may cause fluctuations, making it difficult to achieve convergence. However, the small learning rate may lead to poor results because the models learn data distribution slowly. As shown in Table 4, our approach, TransUnet, achieved the best classification results for lung cancer when the learning rate was set to 0.0001. Besides the learning rate, the batch size was also an important factor that impacted the classification performance of TransUnet. An appropriate batch size would accelerate the convergence speed and improve the classification. Therefore, we conducted four experiments with different batch sizes (8, 16, 32, and 64), which aimed to find the right batch size value. From Table 4, our method had the best accuracy of 0.7697 when the batch size was set to 32. Finally, we also analyzed the effect of optimizers and present the experimental results in Table 4. Usually, we select four popular optimizers, including Adam, Adadelta, Adagrad, and SGD, to learn TransUnet on the LIDI-IDRI. These optimizers have achieved great performance in natural image classification. Clearly, the SGD leads to a performance boost of 0.76–4.29% compared to other optimizers.

In addition to studying condition optimization, we also conducted an experiment to analyze the sensitivity, specificity, and accuracy. As shown in Table 5, TransUnet can produce great classification results, achieving 75.04, 95.93, 87.90, and 0.890% in sensitivity, specificity accuracy, and AUC, respectively. These experiments demonstrated the effectiveness of TransUnet. Meanwhile, we compared TransUnet with the other three classification methods. From Table 6, we can see that the proposed TransUnet had the highest sensitivity and accuracy. Although the specificity was not satisfied, the overall performance of TrasnUnet was better than other methods, because the transformer unit used in TransUnet can extract the general and global features of CT images. In particular, the transformer treats the CT image as a sequence of image patches and extracts the discriminative feature with global self-attention modeling. The global benefit is to classify the lung nodules as malignant or benign. In addition, the Unet was used to decode the feature for classification. The Unet consists of a contracting path to capture context, which enables precise localization for lung nodules. Finally, to implement the classification of CT images, we added the GAP layer to the network to allow each sample to be associated with a classification score. Benefiting from an excellent network structure, TransUnet achieved competitive or superior performance on the testing dataset.

## Conclusion

In this study, we developed a novel TransUnet for differentiating malignant and benign lung nodules. TransUnet used the transformer to extract nodule features and the Unet to decode these features. Finally, the global average pooling was used to differentiate between benign and malignant lung nodules with the deep features of CT images. Based on LIDI-IDRI results, our technique has excellent sensitivity and specificity for classifying lung nodules, which helps assess lung cancer risk in the general population.

## Data availability statement

The original contributions presented in the study are included in the article/Supplementary material, further inquiries can be directed to the corresponding author.

## Author contributions

HW contributed to the conceptualization, study design, data collection, interpretation, and manuscript writing. HZ contributed to data collection, literature search, analysis, fund sourcing, and interpretation. LD contributed to the conceptualization, data analysis, interpretation, writing of the manuscript, and supervision. All authors contributed to the article and approved the submitted version.

## Funding

This work was supported by the Training Program for Young Core Instructors of Henan Universities (2018GGJS137) and the National Natural Science Foundation of China (No. 62172457).

## Conflict of interest

The authors declare that the research was conducted in the absence of any commercial or financial relationships that could be construed as a potential conflict of interest.

## Publisher's note

All claims expressed in this article are solely those of the authors and do not necessarily represent those of their affiliated organizations, or those of the publisher, the editors and the reviewers. Any product that may be evaluated in this article, or claim that may be made by its manufacturer, is not guaranteed or endorsed by the publisher.
